# Mendelian randomisation and experimental medicine approaches to interleukin-6 as a drug target in pulmonary arterial hypertension

**DOI:** 10.1183/13993003.02463-2020

**Published:** 2022-03-10

**Authors:** Mark Toshner, Colin Church, Lars Harbaum, Christopher Rhodes, Sofia S. Villar Moreschi, James Liley, Rowena Jones, Amit Arora, Ken Batai, Ankit A. Desai, John G. Coghlan, J. Simon R. Gibbs, Dee Gor, Stefan Gräf, Louise Harlow, Jules Hernandez-Sanchez, Luke S. Howard, Marc Humbert, Jason Karnes, David G. Kiely, Rick Kittles, Emily Knightbridge, Brian Lam, Katie A. Lutz, William C. Nichols, Michael W. Pauciulo, Joanna Pepke-Zaba, Jay Suntharalingam, Florent Soubrier, Richard C. Trembath, Tae-Hwi L. Schwantes-An, S. John Wort, Martin R. Wilkins, Sean Gaine, Nicholas W. Morrell, Paul A. Corris

**Affiliations:** 1 Dept of Medicine, University of Cambridge, Cambridge, UK; 2 Royal Papworth Hospital, Cambridge, UK; 3 Golden Jubilee Hospital, Glasgow, UK; 4 Heart Lung Research Institute, Imperial College, London, UK; 5 MRC Biostatistical Unit, University of Cambridge, Cambridge, UK; 6 Dept of Epidemiology and Biostatistics, University of Arizona, Tucson, AZ, USA; 7 Dept of Urology, University of Arizona, Tucson, AZ, USA; 8 Dept of Medicine, Indiana University, Indianapolis, IN, USA; 9 Royal Free Hospital, London, UK; 10 Roche Products Limited, Welwyn Garden City, UK; 11 Université Paris-Sud, Le Kremlin-Bicêtre, Paris, France; 12 Royal Hallamshire Hospital, Sheffield, UK; 13 Institute of Metabolic Sciences, University of Cambridge, Cambridge, UK; 14 Division of Human Genetics, Cincinnati Children's Hospital Medical Center, Cincinnati, OH, USA; 15 Royal United Hospital, Bath, UK; 16 Sorbonne Universités, INSERM, Paris, France; 17 Genetics and Molecular Medicine, King's College, London, UK; 18 Mater Misericordiae University Hospital, Dublin, Ireland; 19 Dept of Medicine, Newcastle University, Newcastle, UK; 20 Authors contributed equally to this work; 21 Authors contributed equally to this work

## Abstract

**Background:**

Inflammation and dysregulated immunity are important in the development of pulmonary arterial hypertension (PAH). Compelling preclinical data supports the therapeutic blockade of interleukin-6 (IL-6) signalling.

**Methods:**

We conducted a phase 2 open-label study of intravenous tocilizumab (8 mg·kg^−1^) over 6 months in patients with group 1 PAH. Co-primary end-points were safety, defined by incidence and severity of adverse events, and change in pulmonary vascular resistance. Separately, a mendelian randomisation study was undertaken on 11 744 individuals with European ancestry including 2085 patients with idiopathic/heritable disease for the IL-6 receptor (*IL6R*) variant (rs7529229), known to associate with circulating IL-6R levels.

**Results:**

We recruited 29 patients (male/female 10/19; mean±sd age 54.9±11.4 years). Of these, 19 had heritable/idiopathic PAH and 10 had connective tissue disease-associated PAH. Six were withdrawn prior to drug administration; 23 patients received at least one dose of tocilizumab. Tocilizumab was discontinued in four patients owing to serious adverse events. There were no deaths. Despite evidence of target engagement in plasma IL-6 and C-reactive protein levels, both intention-to-treat and modified intention-to-treat analyses demonstrated no change in pulmonary vascular resistance. Inflammatory markers did not predict treatment response. Mendelian randomisation did not support an effect of the lead *IL6R* variant on risk of PAH (OR 0.99, p=0.88).

**Conclusion:**

Adverse events were consistent with the known safety profile of tocilizumab. Tocilizumab did not show any consistent treatment effect.

## Abstract 2

**Tocilizumab did not block IL-6 signalling in pulmonary arterial hypertension. Multicentre mendelian randomisation studies additionally did not demonstrate evidence for IL-6R in pulmonary arterial hypertension.** https://bit.ly/3xkDxS5

## Introduction

Pulmonary arterial hypertension (PAH) is a rare and often fatal disease characterised by profound remodelling of small pulmonary arteries leading to increased pulmonary vascular resistance (PVR) and right heart failure [1]. Despite existing treatments, mortality remains high and there is a major unmet medical need to identify new therapies. Much interest has focused on the association of PAH with dysregulated immunity, infection and inflammation [2]. Autoimmune diseases are causative in PAH, most commonly scleroderma and systemic lupus erythematosus (SLE). In addition, inflammatory/infectious processes such as HIV infection and schistosomiasis are associated with PAH [3]. Idiopathic and heritable forms of PAH are also associated with autoimmune thyroid disease and human leukocyte antigen subtypes, and the presence of autoantibodies has been noted in up to 93% of patients [4]. Indeed, it has been proposed that idiopathic PAH might be an autoimmune disease [2].

Within the pulmonary vascular lesions of PAH, there is an accumulation of inflammatory cells, including T- and B-lymphocytes with altered regulatory T-cell function [5] and changes in B-cell gene expression [6]. However, it remains unclear whether these findings are a cause or a consequence of disease, *e.g.* caused by exposure to new antigens as a consequence of endothelial damage. One pathway with strong preclinical evidence for a central role in PAH is the interleukin-6 (IL-6) pathway. IL-6 levels are consistently elevated in peripheral blood and within the lung in PAH patients [7]. IL-6 is an independent marker of prognosis, outperforming traditional markers such as PVR and N-terminal pro-brain natriuretic peptide (NT-proBNP) [8]. Increased plasma IL-6 levels and association with the IL-6 receptor (IL-6R) risk alleles are reported in connective tissue diseases (CTDs) known to cause pulmonary hypertension, such as rheumatoid arthritis (RA) [9]. Transgenic overexpression of IL-6 in animal models promotes pulmonary hypertension [10]; conversely, IL-6-deficient mice are protected from hypoxia-induced pulmonary hypertension [11]. Administration of recombinant IL-6 to rats also recapitulates a PAH phenotype [12].

Tocilizumab is an IL-6R antagonist established as safe and effective, primarily in RA [13], and has shown promise in scleroderma [14]. IL-6R antagonism attenuates murine PAH and recent data suggest that ectopic IL-6 signalling directly drives vascular changes in animal models [15]. The rationale for inhibiting IL-6 signalling extends to the direct effects of IL-6 on vascular remodelling in addition to immunomodulation. Isolated case reports have suggested regression of PAH with tocilizumab in associated SLE, mixed CTD and Castleman's disease [16, 17].

We conducted a phase 2 open-label single-arm proof-of-concept study of tocilizumab in PAH. In parallel, we performed a mendelian randomised (MR) case–control study using a single nucleotide polymorphism (SNP) (rs7529229) in IL-6R to infer a causal relationship between IL-6R levels and risk in PAH. This SNP marks a protein quantitative trait locus for circulating IL-6R and includes, in high linkage disequilibrium, a non-synonymous variant (rs8192284) increasing the proteolytic cleavage of the soluble IL-6R from its membrane-bound form [18].

## Methods

### Study design and participants

Patients were recruited across eight centres in the UK (supplementary material) to an open-label single-arm study. The study (TRANSFORM-UK, ClinicalTrials.gov number NCT02676947) [19] was approved by the local research ethics committee (Leicester Central 15/EM/0401).

Patients on stable therapy aged 18–70 years with a diagnosis of group 1 PAH were enrolled: specifically, idiopathic or heritable PAH and PAH associated with CTD excluding SLE, RA and mixed CTD. Selected exclusion criteria included subjects on continuous intravenous (*i.v.*) or subcutaneous prostacyclin infusions, active infection, peripheral blood platelets <100×10^9^ cells·L^−1^, neutrophil count <2×10^9^ cells·L**^−^**^1^, concomitant treatment with biologics, evidence of coronary artery and left heart disease, total lung capacity ≤60% of predicted, forced expiratory volume in 1 s ≤60% of predicted and a 6-min walk test (6MWT) distance <100 m (full inclusion/exclusion criteria can be found in the supplementary material). All participants provided written informed consent.

### Procedures

Participants were given *i.v.* tocilizumab monthly (8 mg·kg**^−^**^1^; Roche Pharmaceuticals) for 6 months on day 1 and weeks 4, 8, 12, 16 and 20 (six doses). Patients attended monthly for infusions and safety data collection. Worsening of PAH was defined by the occurrence of three of the following: a decrease in the 6MWT distance of at least 15% from baseline; the need for additional treatment for PAH; and the worsening of symptoms of PAH, including at least one of a change from baseline to a higher World Health Organization (WHO) functional class and the appearance or worsening of signs of right heart failure unresponsive to oral diuretic therapy. Peripheral blood sampling was undertaken at trial baseline and end of study. Blood was collected for flow cytometric evaluation of leukocyte subsets, RNA sequencing (RNAseq) and serum mediators of inflammation (C-reactive protein (CRP), IL-1β, IL-6, IL-8 and tumour necrosis factor-α (TNF-α)) (supplementary figure S1). Blood was collected in citrate phosphate dextrose adenine and processed within 30 min of sampling after transfer to a lymphocyte separation tube (EZ Lympho-Sep), which was centrifuged as per manufacturer instructions. After a red cell lysis step, peripheral blood mononuclear cells were separated, resuspended in PBS+2 mM EDTA, stored overnight at −80°C and transferred to liquid nitrogen. Immunophenotyping was undertaken on a Fortessa flow cytometer (BD Biosciences). Optimised combinations of primary antibodies were developed based on those proposed by the Human Immunophenotyping Consortium [20]. A minimum of 1×10^6^ events was collected for each analyte. Blood was also processed for cytokine assay in serum separator tubes and Tempus Blood RNA Tubes (3 mL of blood per tube, Thermo Fisher Scientific). Samples were stored at −80°C until completion of the trial. Cytokines were measures from serum samples using a Meso Scale Discovery VPLEX multispot assay platform on the Meso Sector S600 analyser (MSD). Samples were prepared and analysed as per manufacturer instructions (MSD Human Proinflammatory Panel 1- K15049D). RNA extraction was performed using the Tempus Spin RNA Isolation Kit (Thermo Fisher Scientific) and included the optional DNase treatment step using AbsoluteRNA Wash Solution (Thermo Fisher Scientific) in accordance with manufacturer's protocol. Extracted RNA was then concentrated using RNeasy Minelute columns (Qiagen) and eluted in a final volume of 14 μL before depletion of globin mRNA using GLOBINclear (Thermo Fisher Scientific). Final elution of globin-depleted RNA was in 30 μL RNase-free water. The yield and RNA integrity score (RIN) of the samples was determined using the Eukaryote Total RNA Nano Chip Kit (Agilent Technologies) and run on an Agilent 2100 Bioanalyzer (Agilent Technologies); RIN numbers were calculated using the 2100 Expert Software (Agilent Technologies). Libraries for cDNA were prepared with the TruSeq Stranded mRNA Library Prep Kit (Illumina), which generates Poly-A-enriched strand-specific libraries. 1 μg of high-quality RNA was inputted and all protocols were performed following the manufacturer's instructions. Completed libraries were assessed by DNA 1000 Chip (Agilent Biotechnologies) on an Agilent 2100 Bioanalyzer (Agilent Biotechnologies) before normalisation and pooling. Pooled indexed libraries were submitted at 10 nM and sequencing was performed on a HiSeq4000 instrument (Illumina) using a single-end 50 bp run. Gene set analyses were run through cpdb.molgen.mpg.de gene set enrichment database.

### Outcomes

The co-primary end-points were of safety and efficacy, judged respectively on the occurrence of adverse events and serious adverse events as classified by the Medical Dictionary for Regulatory Activities (MedDRA), and change in PVR (PVR Δ) as measured by invasive haemodynamic assessment *via* a fluid-filled right heart catheter using cardiac output measured using the thermodilution technique as per standard techniques previously described [21]. Secondary safety and exploratory efficacy end-points included 6MWT distance as per American Thoracic Society guidelines, Borg Dyspnoea Scale, NT-proBNP, WHO functional class assessment, Cambridge Pulmonary Hypertension Outcome Review (CAMPHOR) assessment, analysis of flow cytometric peripheral blood leukocyte immunophenotyping and serum and plasma measurements of circulating cytokines IL-1β, IL-6, IL-8 and TNF-α. A full description of the study schedule is included in the supplementary material. *Post hoc* descriptive statistics of secondary end-points and disease subtype were pre-specified in the statistical analysis plan.

### Statistical analysis

The sample size (n) powered to detect a 30% reduction in PVR after 6 months of treatment with 90% power and 5% statistical significance was n=17, accounting for a conservative drop-out rate; the minimum target for recruitment was n=21 as published [19]. We present data on modified intention to treat (mITT), defined as the set of patients who have had at least one dose of tocilizumab and at least one post-baseline result, and an intention-to-treat (ITT) analysis as a sensitivity analysis, *i.e.* to assess the existence of potential bias and check that it did not differ from the mITT. Estimates of treatment response rate and associated confidence intervals (or credible intervals and posterior probabilities) are reported. The exception to this is the Wilcoxon signed rank test that was carried out on the PVR fold change as defined in the protocol paper [19]. An additional Bayesian analysis, considered to be more informative of the treatment response of PVR to tocilizumab, was pre-specified using an expert-elicited prior. Any predictor effects on secondary outcomes are reported with confidence intervals. All analyses reported here were performed using the R-Project (version 3.3.3; www.r-project.org). Principal component (PC) analyses of the RNAseq dataset, t-test with Benjamini–Hochberg procedure/false discovery rate (FDR) and penalised regression model (LASSO) using all immunophenotyping variables (cytokines, flow cytometric subsets and RNAseq) as predictors and assessing predictability of response performed best on cross-validation were all undertaken using the R-Project, as were Kaplan–Meier plots.

### Mendelian randomisation

The association of a genetic variant in *IL6R*, rs7529229, with circulating levels of IL-6R was tested in European patients with idiopathic/heritable PAH recruited at Hammersmith Hospital, London, UK. Genotyping were performed through whole genome sequencing in the National Institute for Health Research BioResource study on Rare Diseases (NIHRBR) [22, 23]. Protein measurements were performed through aptamer-based proteomics [24]. The analysis was done with an additive model using natural logarithmic-transformed levels of circulating IL-6R followed by residualisation for age at sampling, gender and population structure (PC1–4) using SNPTEST [25]. The IL-6R protein level association estimate for rs7529229 in patients with idiopathic/heritable PAH was meta-analysed with estimates from two European population-based studies, which applied comparable methodologies [26, 27], using an inverse variance-weighted fixed-effects meta-analysis [28]. A two-sample MR analysis was performed on the meta-analysed estimate for *IL6R* at rs7529229 and accumulated genetic data from international consortia on four idiopathic/heritable PAH­–control comparisons [23]. The causal effect and its standard error were estimated using the Wald ratio as implemented in MR-Base [29]. When rs7529229 was not called in the case–control studies, we used a proxy, rs4537545, in high linkage disequilibrium (r^2^=0.99 in European individuals, run through ldlink.nci.nih.gov).

### Survival analysis

Association of the genetic variant in *IL6R*, rs7529229, with all-cause mortality in idiopathic/heritable PAH patients of the NIHRBR study was used for Kaplan–Meier analysis. Survival analyses were left-truncated for time between diagnosis of PAH and sampling for genotyping.

## Results

**FIGURE 1:**
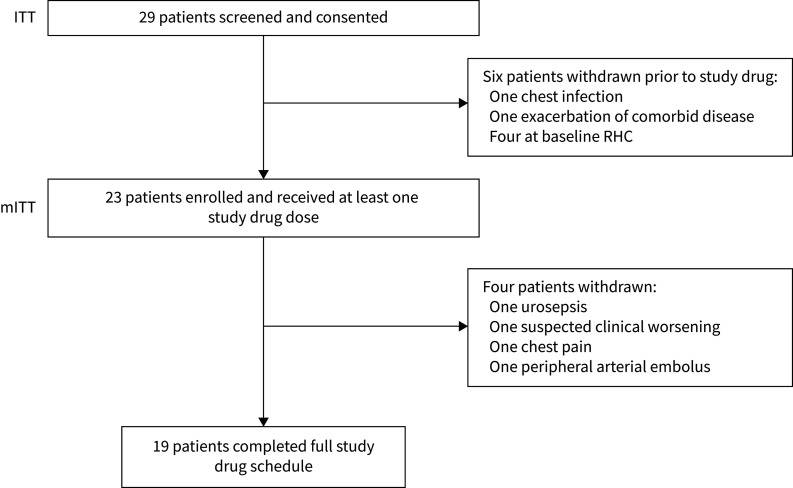
Consort diagram of phase 2 open-label study of tocilizumab. Consort diagram including intention-to-treat (ITT) and modified intention-to-treat (mITT) populations. RHC: right heart catheterisation.

29 patients (male/female 10/19; mean±sd age 54.9±11.4 years) were recruited between January 2016 and April 2017. Of these, 15 patients had idiopathic PAH, 10 had CTD-associated PAH (nine scleroderma, one Sjögren's-associated PAH) and four had heritable PAH (table 1). Six patients were withdrawn prior to drug administration; one owing to a chest infection, one owing to an exacerbation of comorbid disease and four at baseline right heart catheterisation owing to discordant results with the last previous haemodynamic parameters, which the clinician judged non-stable disease as per entry criteria. In total, 23 patients received the study drug (figure 1).

**TABLE 1:** Baseline demographics

**Demographic variable**	**Pooled population**
**Sex M/F (n)**	10/19
**Age years (mean±sd)**	54.9±11.4
**IPAH/FPAH/CTD (n)**	15/4/10
**PVR dyn·s·cm^−5^ (mean±sd)**	612.30±317.63
**mPAP mmHg (mean±sd)**	43.57±11.07
**NT-proBNP pg·mL^−1^ (median (IQR))**	329.50 (549)
**Cardiac output L·min^−1^ (median (IQR** **))**	5.14 (2.18)
**PAH medication (%)**	
Ambrisentan/bosentan/macitentan	17.2/6.9/3.4
Sildenafil/tadalafil/riociguat	58.6/13.8/11.1
**6MWT m (mean±sd)**	425.9±112.1
**WHO class II/III (%)**	48.3/51.7

M: male; F: female; IPAH: idiopathic pulmonary hypertension; FPAH: familial pulmonary hypertension; CTD: connective tissue disease; PVR: pulmonary vascular resistance; mPAP: mean pulmonary arterial pressure; NT-proBNP: N-terminal pro-brain natriuretic peptide; IQR: interquartile range; 6MWT: 6-min walk test; WHO: World Health Organization.

**FIGURE 2:**
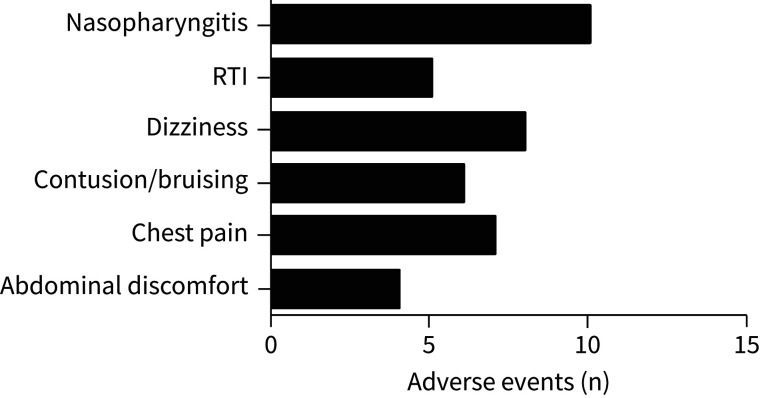
Medical Dictionary for Regulatory Activities (MedDRA)-coded common adverse events. Absolute numbers of MEDRA-coded most common adverse events. RTI: respiratory tract infection.

The drug was discontinued in an additional four patients owing to serious adverse events (figure 1): one urosepsis, one chest pain, one peripheral arterial embolus and one suspected clinical deterioration event that did not fulfil pre-specified protocol criteria for a clinical worsening event on independent adjudication. Vomiting occurred in one patient and was classified as a suspected unexpected serious adverse reaction. There were no deaths. The most common adverse event was nasopharyngitis (figure 2). Adverse events were graded mild (80.0%), moderate (16.6%) and severe (3.4%).

**FIGURE 3:**
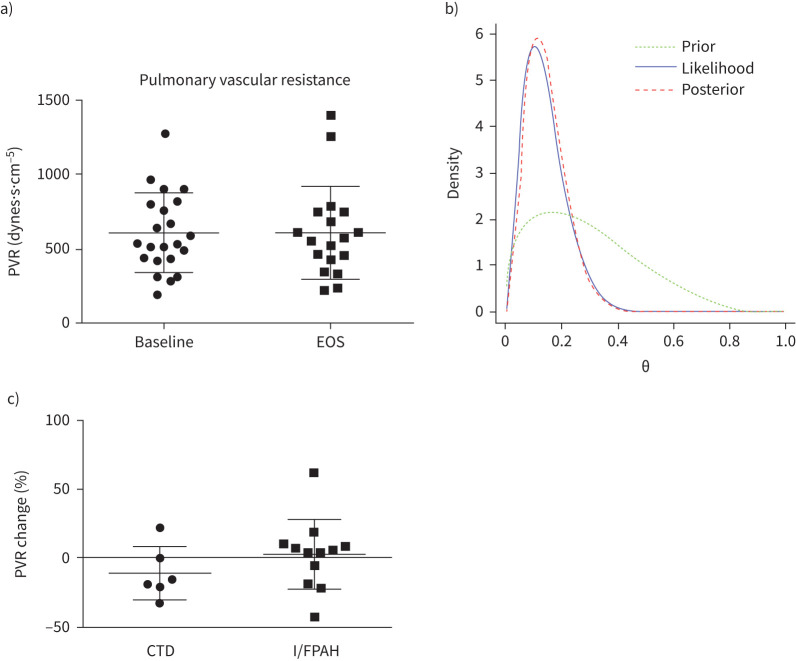
Co-primary end-point; intention-to-treat (ITT) pulmonary vascular resistance (PVR) at baseline and end of study (EOS) in a) all patients (absolute values), b) Bayesian analysis of modified ITT (mITT) and c) mITT percentage change (PVR Δ) by disease group. CTD: connective tissue disease; I/FPAH: idiopathic/familial pulmonary arterial hypertension.

Overall, tocilizumab led to no significant change in PVR from baseline after 6 months of treatment in either the ITT or mITT analysis (figure 3a, b). In *a priori* analyses of PVR stratified by aetiology, four of the six CTD-associated PAH patients receiving study drug had a >15% reduction in PVR *versus* three of 13 patients with idiopathic or heritable PAH (figure 3c).

Bayesian analysis led to the same conclusion as the frequentist analysis (figure 3b). The expert-elicited prior was combined with the observed data to produce a posterior density for the probability of the study drug achieving a PVR change of at least −30%. The posterior probability of the study drug being successful was <1%.

Exploratory secondary end-point analyses were consistent with the primary end-point in that no secondary end-point data clearly supported a treatment effect (table 2). 6MWT distance increased by a mean±sd of 19.1±60.8 m, NT-proBNP increased by a median of 22.5 pg·mL**^−^**^1^ (IQR 275 pg·mL**^−^**^1^) and CAMPHOR decreased by median of 2 (IQR 10). WHO functional class changed in six patients, improving in four patients and deteriorating in two.

**TABLE 2:** Secondary end-points

**Secondary end-points**	**Pooled population**
**6MWT Δ m (mean±sd)**	19.1±60.8
**NT-proBNP Δ pg·mL**^−1^ **(median (IQR))**	22.5 (275)
**Bord score Δ (mean±sd)**	0.05±0.05
**CAMPHOR Δ (median (IQR))**	−2 (10)
**WHO class:**	
Unchanged/improved/deteriorated (n)	17/4/2
IL-6 Δ pg·mL^−1^ (median (IQR))	9.5 (7.3)
CRP Δ mg·L^−1^ (median (IQR))	−1.2 (2.2)

6MWT: 6-min walk test; NT-proBNP: N-terminal pro-brain natriuretic peptide; IQR: interquartile range; Borg: Borg Rating of Perceived Exertion; CAMPHOR: Cambridge Pulmonary Hypertension Outcome Review (patient-reported outcome measure); WHO: World Health Organization; IL-6: interleukin-6; CRP: C-reactive protein.

Consistent with effective inhibition of IL-6 signalling, tocilizumab exposure increased plasma IL-6 by a median of 9.5 pg·mL**^−^**^1^ (IQR 7.3 pg·mL**^−^**^1^) and CRP decreased by a median of 1.2 mg·L**^−^**^1^ (IQR 2.2 mg·L**^−^**^1^) (table 2 and supplementary figure S1). Plasma levels of IL-1β, IL-8 and TNF-α did not change (supplementary figure S1). In addition, immunophenotyping of peripheral blood cells did not show any significant changes in either B- or T-cell populations (supplementary figure S2). In RNAseq analyses there were no significant single gene expression changes after correction for multiple comparisons in response to tocilizumab. Network analyses of gene set over-representation and ontological analyses demonstrated changes in immune-mediated processes (supplementary table S1). Enriched protein complex-based sets were also dominated by signals associated with T- and B-cell-mediated immunity, notably the B-cell receptor (supplementary table S2).

PC analyses of RNAseq gene expression and expression changes did not discriminate either CTD-associated PAH or the potential responders in the cohort (t-test, Benjamini–Hochberg procedure with FDR<0.1) (supplementary figure S3). Overall, none of the immunophenotyping data correlated with treatment response or predicted responders from non-responders either individually or using integrative modelling. Consistent with a treatment effect on gene expression, in PC analysis there was modest evidence that pre- and post-treatment categorisation could be differentiated in two dimensions on the basis of the first two PCs (Peacock test, p=0.033). In a penalised regression model (LASSO) using all immunophenotyping variables as predictors and assessing predictability of response, the model performed best on cross-validation when no variables were included in the predictive model (supplementary figure S4). Therefore, there was no additional value added by analyses that utilised all of the inflammatory dataset, and no inflammatory marker predicted treatment response either individually or in combinatorial modelling.

**FIGURE 4:**
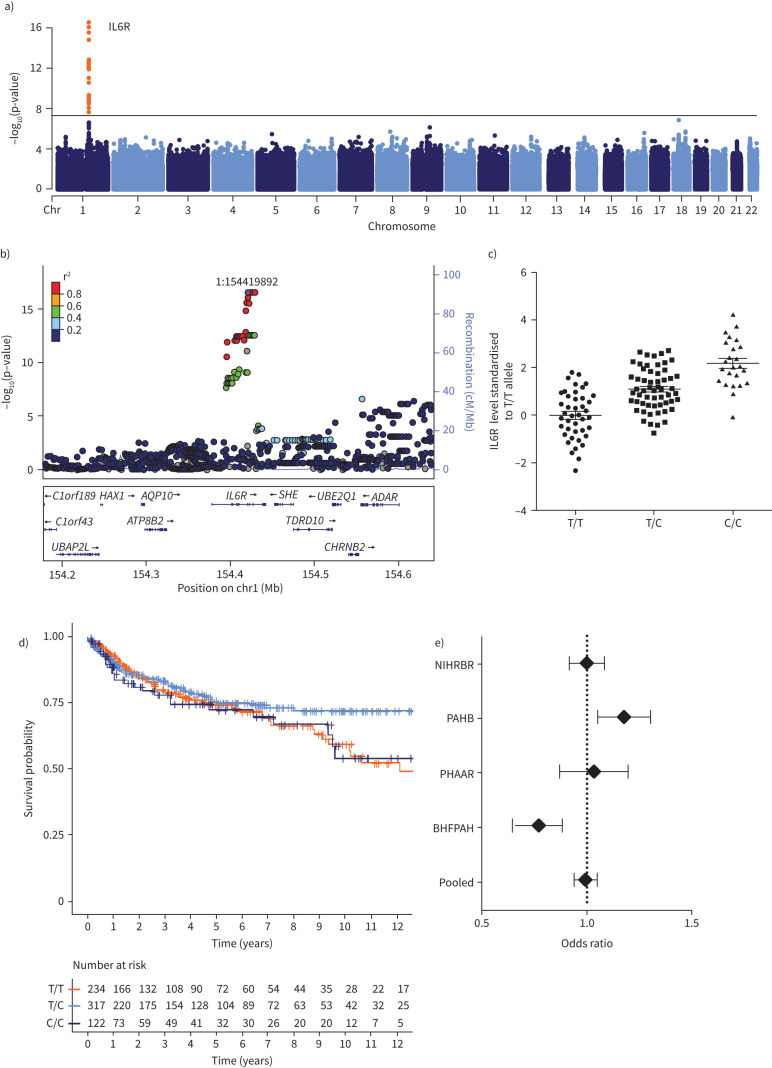
Mendelian randomisation of interleukin-6 receptor (IL-6R) in idiopathic pulmonary arterial hypertension (PAH). a) Protein quantitative trait locus (pQTL) in genome-wide association studies. b) Locus zoom of pQTL. c) Circulating IL-6R levels performed through aptamer-based protein measurements by genotype of *IL6R* single nucleotide polymorphism (SNP) rs7529229 (T/T, T/C and minor allele C/C) in UK patients with familial/idiopathic PAH. d) Left-truncated Kaplan–Meier plots for patients with PAH stratified by *IL6R* SNP rs7529229 in the UK National Institute for Health Research BioResource Rare Diseases study (NIHRBR) study cohort. e) Forest plot meta-analyses of association *IL6R* lead SNP association with familial/idiopathic PAH in international studies. PAHB: US National Biological Sample and Data Repository for Pulmonary Arterial Hypertension (also known as PAH Biobank); PHAAR: Pulmonary Hypertension Allele-Associated Risk study; BHFPAH: British Heart Foundation Pulmonary Arterial Hypertension study.

In an association analysis, each minor allele, “C”, at rs7529229 was associated with an increase in circulating IL-6R level in 124 patients with idiopathic or heritable PAH (effect estimate 0.27±0.03, p=2.69×10^−16^, figure 4c). A meta-analysis including these PAH patients and an additional 4299 individuals from two population-based cohorts revealed an overall effect estimate of 0.81±0.01 per “C” allele (OR 2.1, p<1×10^−300^) but no trans-acting locus associated with circulating IL-6R (figure 4a, b). MR revealed no significant association of rs7529229 with risk of PAH in our pooled analyses of 2085 cases with idiopathic or heritable PAH and 9659 controls (OR 0.99, 95% CI 0.9–1.09, p=0.88, figure 4e). In addition, genotype at *IL6R* SNP rs7529229 was not associated with all-cause mortality in the NIHRBR study cohort (figure 4d).

## Discussion

This proof-of-concept open-label clinical trial demonstrated no significant effect of tocilizumab in prevalent group 1 PAH. This was despite evidence that the drug led to expected changes in biomarkers of target engagement: increased IL-6 and decreased CRP levels. In addition, B- and T-cell activation pathways were the top ontology hits in RNAseq analysis. It is possible that the stable prevalent patients recruited to this study were less likely to have active inflammation, although the IL-6 and CRP levels measured are in line with previous reports [8].

The lack of efficacy in this trial would seem to contradict the extensive data in preclinical cell and animal models for an important potentially causal role for IL-6. There are a number of explanations for this. It is possible that inflammation is a response to vascular injury rather than a causal factor in human PAH, though in MR studies we did not see an association with disease course based on mortality. It is notable that in PAH cohorts the IL-6 levels are skewed with a non-normal distribution and the majority of patients do not have significantly higher levels [8]. This raises the possibility that disease is driven by inflammation only in a subset of patients, or that there are flares of inflammatory activity in patients. Meta-analyses of autoimmune phase 3 trials stratified by IL-6 demonstrate disappointing correlations with clinical response [30] and this approach is not recommended in any of the current indications for IL-6 receptor antagonism. Our planned sub-analyses of response using extensive inflammatory markers, including IL-6, suggest again that stratifying patients by IL-6 levels would not have been of additional value. We note that in our original report, IL-6 levels were almost double those reported here [8]. Though no trial has strongly suggested stratification by IL-6 works in other diseases, this may reflect a random sampling bias, the effect of targeted therapies, differences in assays or the requirements for stable disease, which one can hypothesise negatively selects against patients with active inflammation.

Our entry criteria for CTD-associated PAH excluded specific, less common autoimmune patient groups with RA, SLE and mixed CTD. The reasons for exclusion included that these patients are already receiving immunosuppression and it was not felt appropriate from a safety perspective for a first trial of immunosuppression to include subsets of patients on multiple therapies. In addition, this would have led to greater heterogeneity in the patient cohort. Despite this, there was a reduction in PVR in four of six patients with CTD-associated PAH. Immunosuppression is widely used in the context of CTD but at present the effects on the associated PAH have not been investigated in randomised controlled trials. Based on the limited data presented here, it may be advisable to prioritise future trials of immunosuppression in CTD-associated PAH. This will be complicated by the heterogeneous nature of both the patient population and the use of immunosuppressive agents in some patients prior to the onset of PAH. Such trials would need to consider the mixed disease population and mixed background of immunosuppressive drugs carefully.

Potential limitations of our experimental study are the small size and open-label design. In addition, it is possible that the impact of immunosuppression on PVR, if present, could take longer than 6 months. Previous meta-analyses have demonstrated that PVR is not a placebo-responsive end-point [19]. This is supported by our trial results. In previous trials of a similar duration, PVR was more likely to deteriorate in placebo arms and it is therefore possible that we have missed a difference between active drug and placebo, had we included one. Nevertheless, this difference would by definition have been small and of limited clinical utility in the context of the additional risk patients are exposed to by immunosuppression.

This is the first time in PAH where genetic data have been used in tandem to test drug target validity in combination with early phase go/no-go investigator-led clinical trials. Our association analysis results confirm that rs7529229 associates with circulating IL-6R levels, as anticipated. However, there was no significant association between that variant and the presence or absence of PAH, providing additional evidence that modulation of IL-6 signalling may not be of benefit for most PAH patients. If the approach of leveraging genetic data in evaluating therapies gains traction, the possible signal in CTD/scleroderma is potentially supported by existing meta-analyses data in scleroderma not stratified by presence of PAH, where STAT3 emerges as a risk loci [31]. Such approaches will be a potentially useful addition to the toolkit for drug development. Coupling fast and statistically efficient trial designs to MR studies is feasible even in rare disease, where co-operative international genetics consortia are making open access genomic data a reality in numbers robust enough to test hypotheses. We acknowledge that our MR meta-analyses are not powered to pick up smaller effects and this still cannot be ruled out.

In summary, treatment with tocilizumab is feasible in PAH but demonstrated no significant effects on haemodynamic parameters or exploratory secondary end-points in heritable or idiopathic PAH. MR studies were in line with these results. A potential improvement was noted in the small subgroup of patients with CTD-associated PAH and this warrants further investigation. Future trials in immunomodulation in PAH need to consider whether endotyping and stratification of patients earlier in the disease process might be worthwhile.

## Supplementary material

10.1183/13993003.02463-2020.Supp1 **Please note:** supplementary material is not edited by the Editorial Office, and is uploaded as it has been supplied by the author.
Supplementary material ERJ-02463-2020.Supplement

## Shareable PDF

10.1183/13993003.02463-2020.Shareable1 This one-page PDF can be shared freely online.
Shareable PDF ERJ-02463-2020.Shareable
