# “Mendelian randomisation and experimental medicine approaches to interleukin-6 as a drug target in pulmonary arterial hypertension.” Mark Toshner, Colin Church, Lars Harbaum, *et al*. *Eur Respir J* 2022; 59: 2002463.

**DOI:** 10.1183/13993003.52463-2020

**Published:** 2022-07-07

One of the author names was presented incorrectly in the published version of this article. The author “Sofia S. Villar Moreschi” should have been correctly presented as “Sofia S. Villar”.

The article has been corrected and republished online.

