# Supplementary material for: Mendelian randomisation and experimental medicine approaches to interleukin-6 as a drug target in pulmonary arterial hypertension
Source: Eur Respir J. 2022 Mar 10;59(3):2002463. doi: 10.1183/13993003.02463-2020 (PMC8907935; doi:10.1183/13993003.02463-2020)
Supplement: Supplementary file 1 [file ERJ-02463-2020.Supplement.pdf]

Supplementary Appendix for

Mendelian randomization and tocilizumab phase 2 trials do not to support IL-6 as a drug target in group 1 pulmonary arterial hypertension

Page 2: UK phase 2 clinical trial participating centres

Page 2: Supplementary methods

Page 5: Supplementary tables

Page 6: Supplementary figures

Page 9: Phase 2a clinical trial visit schedule

Page 10 Study Cohorts used for Mendelian Randomisation

Page 11: Full authorship and affiliations details

UK Phase 2 clinical trial participating centres
Freeman Hospital, Newcastle
Golden Jubilee Hospital, Glasgow
Hammersmith Hospital, London
Royal Brompton Hospital, London
Royal Free Hospital, London
Royal Hallamshire, Sheffield
Royal Papworth Hospital, Cambridge
Royal United Hospital, Bath

Supplementary methods
Inclusion/ Exclusion criteria
Inclusion
- Subject must be between 18 and 70 years of age, inclusive, at the Screening visit
- Subject must weigh >40 kg at the Screening Visit.

PAH Diagnosis and Classification
- Subjects must have a diagnosis of group 1 PAH due to the following:
    - Idiopathic or Heritable PAH
    - PAH associated with connective tissue disease excluding SLE, RA, mixed CTD (e.g. limited scleroderma, diffuse scleroderma, or overlap syndrome)
    - Drug or toxins
- Subject must have a current diagnosis of being in WHO Functional Class II-IV.
- Subject must meet all of the following haemodynamic criteria by means of a RHC prior to screening:
    - mPAP of ≥25 mmHg
    - PVR ≥ 300 dynes/sec/cm5
    - PCWP or LVEDP of ≤12 mmHg if PVR ≥300 to <500 dyne/sec/cm5, or
    - PCWP/LVEDP <15 mmHg if PVR ≥500 dynes/sec/cm5
- Subject must meet all of the following pulmonary function tests completed no more than 24 weeks before the Screening visit: Total lung capacity (TLC) ≥60% of predicted normal and Forced expiratory volume in one second (FEV1) ≥60% of predicted normal
- Subjects are required to have a documented negative V/Q scan or pulmonary arteriogram confirming the absence of CTEPH prior to screening.
- Subject must walk a distance of ≥100m and ≤500m at the screening visit.
- Subject, with or without supplemental oxygen, must have a resting arterial oxygen saturation (SaO2) ≥85% as measured by pulse oximetry at the Screening Visit.
- Female subject of childbearing potential, if sexually active, must agree to use 2 reliable methods of contraception from the Screening Visit until study completion and for at least 30 days following the last dose of Investigational Product. Subjects who have had a Copper T 380A IUD or LNg 20 IUD inserted are not required to use additional methods of contraception.
- Subject must agree not to participate in a clinical study involving another investigational drug or device throughout this study.
- Subject must be competent to understand the information given in the Institutional Review Board (IRB) or Independent Ethics Committee (IEC) approved Informed Consent Form and must sign the form prior to the initiation of any study procedures.

- Subject must be stable on an unchanged PAH therapeutic regime for at least 1 month prior to screening.

Exclusion Criteria

Subjects meeting any of the following criteria must not be enrolled in the study

PAH Treatments

- Subjects on continuous infusions either intravenously or subcutaneously.
- Subject has a known hypersensitivity to the Investigational Products, the metabolites, or formulation excipients.
- Subject has severe renal impairment (creatinine clearance <30 mL/min) at the Screening Visit

Medical History/Current Medical Conditions

<u>Liver</u>

- Subject has severe hepatic impairment (Child-Pugh class C with or without cirrhosis) at the Screening Visit.

Haematology and bleeding disorders

- Subject has clinically significant anaemia in the opinion of the investigator, in particular from pyruvate kinase and G6PD deficiencies.
- Subjects with bleeding disorders or significant active peptic ulceration in the opinion of the investigator.
- Subject has peripheral blood platelets $<100x10^{9}$/L.
- Subject has a neutrophil count $<2x10^{9/}$L.

Cardiovascular

- Subject has had an acute myocardial infarction within the last 90 days prior to screening

Subjects must not have 3 or more of the following left ventricular disease/dysfunction risk factors:

- Body Mass Index (BMI) ≥ 35
- Historical evidence of significant coronary disease established by any one of:
  - history of myocardial infarction
  - history of percutaneous intervention
  - angiographic evidence of CAD (>50% stenosis in at least one vessel), either by invasive angiography or by CT Angiography
  - positive stress test with imaging (either pharmacologic or with exercise)
  - previous coronary artery surgery
  - chronic stable angina

Ophthalmic

- Subject has a past medical history of macular degeneration or visual field defect of any cause.
- Subject has a hereditary degenerative retinal disorder (e.g. retinitis pigmentosa).

General Medical Conditions

- Subject with cardiovascular, liver, renal, haematologic, gastrointestinal, immunologic, endocrine, metabolic, or central nervous system disease that, in the opinion of the Investigator, may adversely affect the safety of the subject and/or efficacy of the investigational product or severely limit the lifespan of the subject other than the condition being studied.
- Subject has a history of malignancies within the past 5 years, except for a subject with localized, non-metastatic basal cell carcinoma of the skin, in situ carcinoma of the cervix, or prostate cancer who is not currently or expected, during the study, to undergo radiation therapy, chemotherapy, and/or surgical intervention, or to initiate hormonal treatment.

General Criteria

- Female subject who is pregnant or breastfeeding.
- Subject has demonstrated noncompliance with previous medical regimens.
- Subject has a recent (within 1 year) history of abusing alcohol or illicit drugs.
- Subject has participated in a clinical study involving another investigational drug or device within 4 weeks before the Screening Visit.

Appendix Table S1 RNAseq pathway and enrichment analyses

complex name	set size	candidates contained	p-value	q-value
mIg	47	6 (12.8%)	7.84e-06	4.52e-05
BCR complex	49	6 (12.2%)	1e-05	4.52e-05
S1P/S1P3/G12/G13	3	2 (66.7%)	0.000313	0.000705
TGAV-P2RY2-GNA12 complex	3	2 (66.7%)	0.000313	0.000705

Enriched protein complex-based sets.

Appendix Table S2 Enriched gene ontology-based sets

Gene ontology terms	Set size	Candidates contained	P value	Q value
GO:0002443 leukocyte mediated immunity	867	22 (2.5%)	0.000103	0.0146
GO:0006887 exocytosis	880	22 (2.5%)	0.000126	0.0146
GO:0001882 nucleoside binding	391	13 (3.3%)	0.000229	0.0142
GO:0045321 leukocyte activation	1258	27 (2.2%)	0.000266	0.0166
GO:0001775 cell activation	1408	29 (2.1%)	0.00031	0.0166
GO:0002252 immune effector process	1237	26 (2.1%)	0.000478	0.017
GO:0043299 leukocyte degranulation	538	15 (2.8%)	0.000512	0.0357
GO:0002433 immune response-regulating cell surface receptor signaling pathway involved in phagocytosis	138	7 (5.1%)	0.000612	0.0357

Appendix Figure S1 Serum change in inflammatory mediators

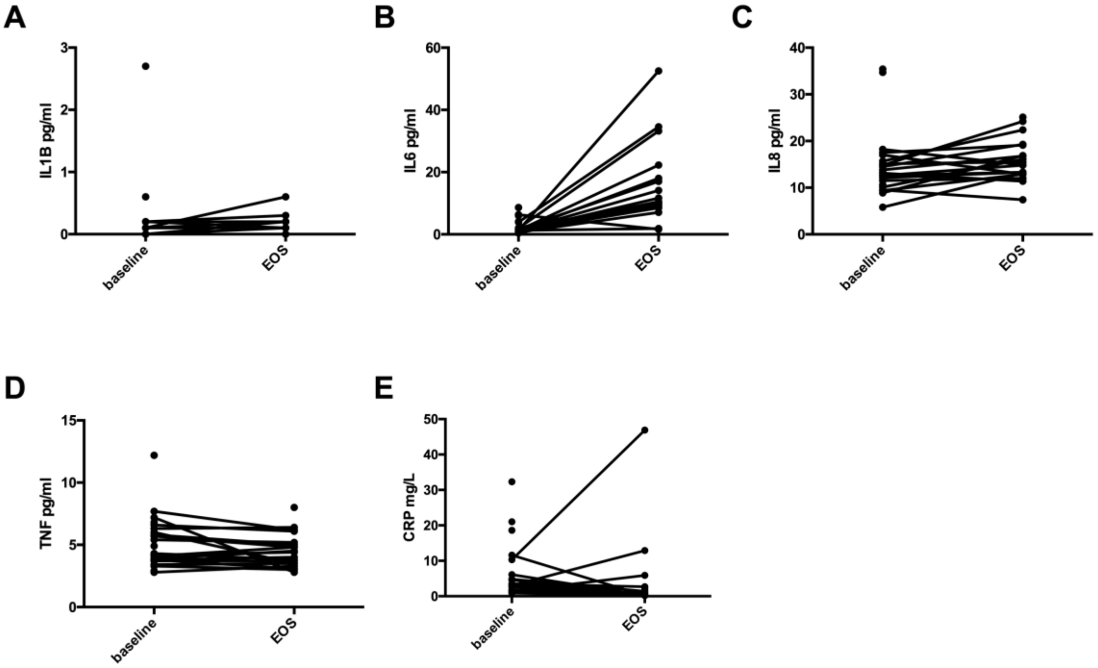

Changes in serum cytokines A) Interleukin-1β B) Interleukin-6 C) Interleukin-8 D) Tumour necrosis factor-α E) C reactive protein.
EOS- end of study

Appendix Figure S2 Immunophenotyping of B and T cell subsets by flow cytometry

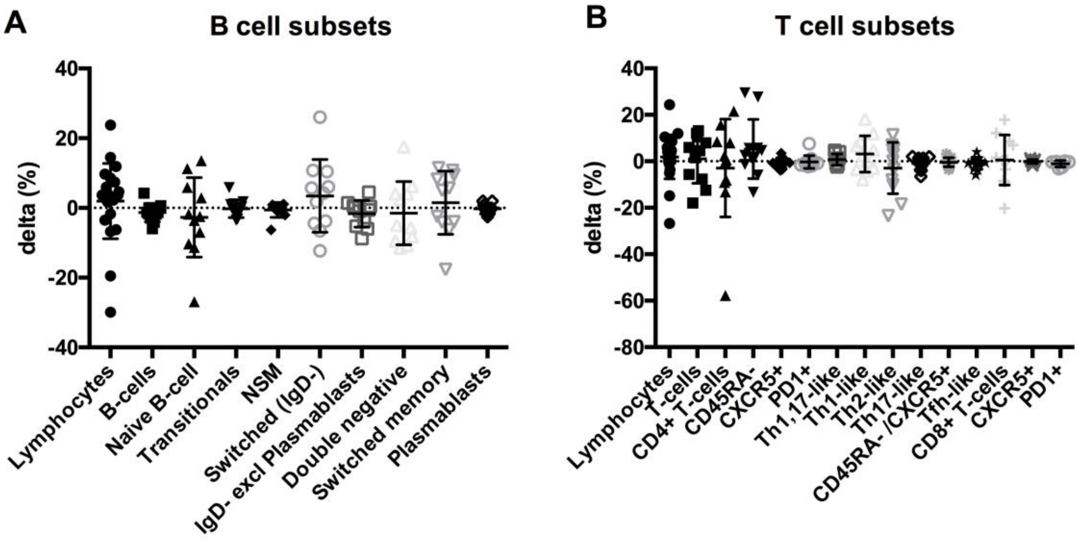

Flow cytometric evaluation of change in lymphocyte subsets from baseline to end of study in A) B cell and subsets and B) T cells and subsets

Appendix Figure S3 Principal Component Analyses of RNAseq data from peripheral blood

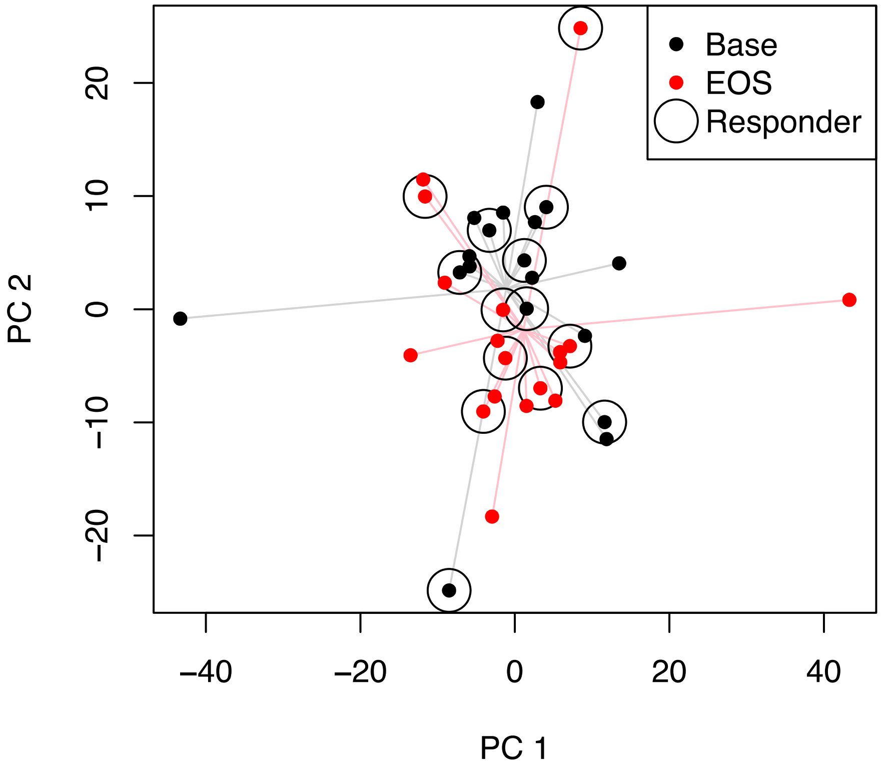

Principal component scores for samples by timepoint. The two measurements for a given individual are reflections in the x-y line. Lines are drawn from each point to the mean of points of that colour. Base (baseline); EOS (end of study)

Appendix Figure S4 Penalised regression model (LASSO)

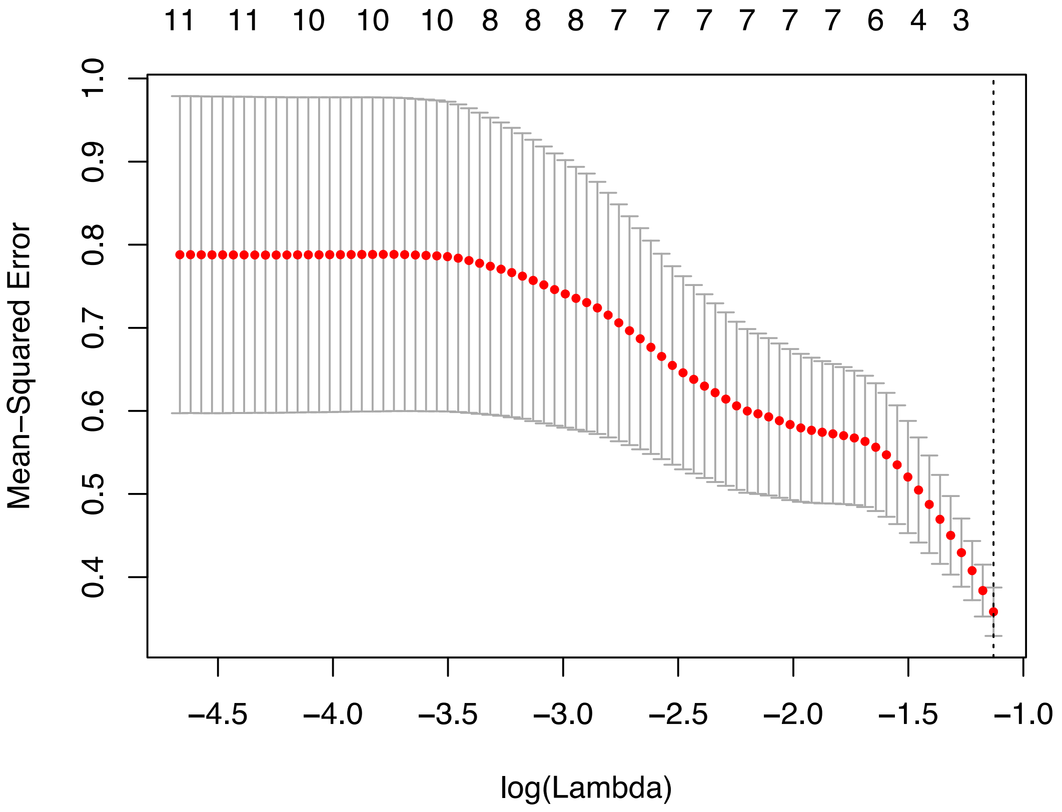

Cross-validated error in lasso model by Lambda value. Higher lambda corresponds to fewer variables included. The error was lowest at maximal lambda, with no variables included at all.

Table S3. Study assessments and procedures

PERIODS	Name	SCREENING	TREATMENT*			PREMATURE STUDY TERMINATION	SAFETY FOLLOW UP
	Duration	Up to 4 weeks	24 Weeks				30 DAYS
VISITS	Number	1	2	3 ,4, 5, 6,7	8		
	Name	Screening	Baseline	Week, 4, 8, 12, 16 + 20 Unscheduled	Week24 EOS	Premature Termination	Safety Follow up
	Time	Day-30 to Day 1	Day1	Visits +-3days	Day ±3 days	Visit within 7 days of premature termination	30 days after drug discontinuation
Inclusion/exclusion		X					
Informed consent		X					
Medical History		X					
Demographics		X					
Urine Pregnancy Test		X	X	X	X	X	
Physical Exam		X	X	X	X	X	X
Who Class		X	X	X	X	X	X
Vital Signs		X	X	X	X	X	X
Pulmonary Function Tests		X					
RHC			X		X	X	
6MWT/Borg index		X	X	X	X	X	
QOL questionnaire			X	X	X	X	
12-lead ECG			X		X	X	
AEs/SAEs			X	X	X	X	X
Conmeds			X	X	X	X	X
PAH meds		X					
ProBNP			X	X	X	X	
Routine Labs		X	X	X	X	X	X
Immunophenotyping			X		X	X	
Cytokines			X		X	X	
IV infusion			X	X			

Study Cohorts used for Mendelian Randomisation

As described in reference 23 (Rhodes et al) the meta-analyses data were taken from the following cohorts. UK National Institute of Health Research BioResource (NIHRBR) for Rare Diseases study – PAH was defined by right heart catheterization measurements including mean pulmonary artery pressure (mPAP) > 25 mmHg, pulmonary capillary wedge pressure (PCWP) < 15 mmHg, and pulmonary vascular resistance (PVR) > 3 Woods Units. Eligible cases were recruited from the UK National Pulmonary Hypertension Centres, as well as Université Sud Paris (France), the VU University Medical Center Amsterdam (The Netherlands), the Universities of Gießen and Marburg (Germany), and San Matteo Hospital, Pavia (Italy). Study recruitment was undertaken between 29 Jan 2003 and 4 Jan 2017, and patients were followed up to 24 Mar 2017. Cases were excluded if they were not able to provide written informed consent or were diagnosed with other forms of PAH. One patient withdrew from the study. Controls consisted of patients with other rare diseases from the NIHRBR rare disease study
.
US National Biological Sample and Data Repository for Pulmonary Arterial Hypertension/PAH Biobank (PAHB) study - PAH was defined by right heart catheterization measurements including mPAP > 25 mmHg, PCWP < 18 mmHg, and PVR > 2.5 Woods Units. Eligible cases were recruited from 29 pulmonary hypertension centers across the United States and enrolled as part of the National Biological Sample and Data Repository for Pulmonary Arterial Hypertension (PAH Biobank, www.pahbiobank.org) funded by the National Institutes of Health/National Heart Lung and Blood Institute (R24HL105333). PAH cases were recruited between October 3, 2012 to March 14, 2016. Controls were selected from the Vanderbilt Electronic Systems for Pharmacogenomic Assessment (VESPA) cohort2-4 ascertained at Vanderbilt University (VU). The VESPA project used BioVU, VU's large DNA repository coupled to de-identified data from electronic health records (EHRs), to investigate the genetic component of individual response to medications for 28 pharmacogenomic phenotypes unrelated to PAH, including angiotensin-converting enzyme (ACE) inhibitor-induced cough, vancomycin-induced kidney dysfunction, heparin-induced thrombocytopenia, and others2. BioVU was approved by the Institutional Review Board at Vanderbilt University as described previously5. BioVU recruited using an opt-out model until January 2015, at which time an opt-in model was adopted. The complete VESPA project population includes 11,639 genotyped individuals from BioVU (84% Caucasian and 12% African American) with a median age of 61.6 years. Only samples of European descent that were genotyped on the Illumina® Omni5-Quad BeadChip array were included in the control population for this study (n=2,144). The controls include individuals with type 1 diabetes (n=251) and connective tissue diseases (n=56). 31 controls with a diagnosis of pulmonary hypertension were excluded. Combining cases with controls data (n=2,144), a total of 4,245 subjects were available for analysis.

Paris Pulmonary Hypertension Allele-Associated Risk cohort (PHAAR) study - Diagnosis with PAH was defined by hemodynamic measurement during right-heart catheterization for all cases identified by the French PAH Network between 1 January, 2003, and 1 April, 2010. For all cases, PAH was defined as a mPAP >= 25 mmHg associated with normal PCWP. Cases with known pathogenic mutations in BMPR2 or ACVRL1 were excluded. Further details have been published7. The control group was composed of a random sample of 1,140 subjects who were free of any chronic disease from the 3C Study8. The 3C Study is a population-based prospective cohort with a 4-year follow-up carried out in three French cities: Bordeaux (southwest France), Montpellier (southeast France) and Dijon (central eastern France).

British Heart Foundation Pulmonary Arterial Hypertension GWAS (BHFPAH) study - The BHFPAH cohort comprised IPAH patients recruited from the Pulmonary Hypertension Division at University Hospital Giessen or from specialist PAH centres in the UK, namely Royal Hallamshire Hospital Pulmonary Vascular Unit, Northern Pulmonary Vascular Unit – Freeman Hospital, Papworth Hospital NHS Foundation Trust, National Pulmonary Hypertension Service – Hammersmith Hospital, Royal Brompton and Harefield NHS Foundation Trust, and the Scottish Pulmonary Vascular Unit. Patients were recruited between 3 Dec 1998 and 1 Dec 2011 and all provided written informed consent to participate in the study. The BHFPAH control cohort was population based, comprising of individuals ascertained through the Wellcome Trust Case Control Consortium, UK or recruited as part of the Food Chain Plus (FoCus) cohort, Germany. Individuals in

the Focus cohort with BMI>30 were excluded. All IPAH cases and UK controls were genotyped at King’s College London on an Illumina HiScan system, whilst genotype data for the German controls were generated by PopgenFull list of collaborator and affiliation details:

Full authorship and affiliations details

NIHR BioResource – Rare Disease Consortium, US PAH Biobank Consortium, Marta Bleda [1], Matthias Haimel [1,2,3], Laura Southgate [4,5], Marine Germain [6], Michael W. Pauciulo, B.S [7], Charaka Hadinnapola [1], Jurjan Aman [8], Barbara Girerd [9], Amit Arora, MD MPH [10], Jo Knight, Ph.D. [11], Ken B. Hanscombe [5], Jason H. Karnes [12], Marika Kaakinen, Ph.D. [8], Henning Gall [13], Anna Ulrich [8], Inês Cebola [14], Jorge Ferrer [14], Katie Lutz [7], Emilia M. Swietlik [1], Ferhaan Ahmad [15], Philippe Amouyel [16], Stephen L. Archer [17], Rahul Argula [18], Eric D. Austin [19], David Badesch [20], Sahil Bakshi [21], Christopher F. Barnett [22], Raymond Benza [23], Nitin Bhatt [24], Harm J. Bogaard [25], Charles D. Burger [26], Murali M. Chakinala [27], Martin Johnson [28], Robin Condliffe [29], James Lordan [30], Cesare Danesino [31,32], Stéphanie Debette [33], C. Gregory Elliott [34], Jean Elwing [35], Melanie Eyries [6], Terry Fortin [36], Andre Franke [37], Robert P. Frantz [38], Adaani Frost [39], Joe G. N. Garcia [40], Stefano Ghio [32], Hossein-Ardeschir Ghofrani [13,8], John B. Harley [41,74], Hua He [7], Nicholas S. Hill [42], Russel Hirsch [43], Arjan C. Houweling [25], Dunbar Ivy [44], James Klinger [45], Gabor Kovacs [46,47], Tim Lahm [48], Matthias Laudes [49], Allan Lawrie [29,73], Rajiv D. Machado [50], Robert V. MacKenzie Ross [51], Keith Marsolo [52], Lisa J. Martin [7], Shahin Moledina [53], David Montani [9], Steven D. Nathan [54], Michael Newnham [1], Andrea Olschewski [46], Horst Olschewski [46,47], Ronald J. Oudiz [55], Willem H. Ouwehand [2,3], Andrew J. Peacock [28], Inga Prokopenko [8], Zia Rehman [56], Ivan M. Robbins [57], Dan M. Roden [58,59], Erika B. Rosenzweig [60], Ghulam Saydain [58], Laura Scelsi [32], Robert Schilz [62], Werner Seeger [13], Christian M. Shaffer [57], Robert W. Simms [63], Marc Simon [64], Olivier Sitbon [9], Mark Southwood [65], Haiyang Tang [40,75], Alexander Y. Tchourbanov [66], Thenappan Thenappan [67], AA Roger Thompson [29], Fernando Torres [68], Carmen M. Treacy [1], David-Alexandre Trégouët [6], Anton Vonk Noordegraaf [25], Quinten Waisfisz [25], Anna K. Walsworth [7], Robert E Walter [69], John Wharton [8], R. James White [70], Jeffrey Wilt [71], Delphine Yung [72],

[1] Department of Medicine, University of Cambridge, Cambridge, United Kingdom;
[2] Department of Haematology, University of Cambridge, Cambridge, United Kingdom;
[3] NIHR BioResource - Rare Diseases, Cambridge, United Kingdom;
[4] Molecular and Clinical Sciences Research Institute, St George's University of London, London, United Kingdom;
[5] Division of Genetics and Molecular Medicine, King's College London, London, United Kingdom;
[6] Sorbonne Universités, UPMC Univ. Paris 06, Institut National pour la Santé et la Recherche Médicale (INSERM), Unité Mixte de Recherche en Santé (UMR_S) 1166, Team Genomics & Pathophysiology of Cardiovascular Diseases; ICAN Institute for Cardiometabolism and Nutrition, Paris, France;
[7] Division of Human Genetics, Cincinnati Children's Hospital Medical Center, Department of Pediatrics, University of Cincinnati College of Medicine, Cincinnati OH, United States;
[8] Centre for Pharmacology & Therapeutics, Department of Medicine, Hammersmith Campus, Imperial College London, London, United Kingdom;
[9] Université Paris-Sud, Faculté de Médecine, Université Paris-Saclay; AP-HP, Service de Pneumologie, Centre de référence de l’hypertension pulmonaire, Hôpital Bicêtre, Le Kremlin-Bicêtre; INSERM UMR_S 999, Hôpital Marie Lannelongue, Le Plessis Robinson, Paris, France;
[10] Division of Urology, Department of Surgery, The University of Arizona College of Medicine, Tucson, AZ, United States;

[11] Data Science Institute, Lancaster University, Lancaster, United Kingdom;
[12] Department of Pharmacy Practice & Science, University of Arizona College of Pharmacy, Sarver Heart Center and Center for Applied Genetics and Genomic Medicine (TCAG2M), University of Arizona College of Medicine, Tucson, AZ, United States;
[13] University of Giessen and Marburg Lung Center (UGMLC), member of the German Center for Lung Research (DZL) and of the Excellence Cluster Cardio-Pulmonary System (ECCCPS), Giessen, Germany;
[14] Section of Epigenomics and Disease, Department of Medicine, Hammersmith Campus, Imperial College London, London, United Kingdom;
[15] Division of Cardiovascular Medicine, University of Iowa, Iowa City IA, United States;
[16] Univ. Lille, Inserm, CHU Lille, Institut Pasteur de Lille, U1167 - RID-AGE - Risk Factors and Molecular Determinants of Aging-related Diseases, F-59000, Lille, France;
[17] Queen's University, Kingston ON, Canada;
[18] Medical University of South Carolina, Charleston SC, United States;
[19] Vanderbilt University-Peds, Nasville TN, United States;
[20] University of Colorado Denver, Aurora CO, United States;
[21] Baylor Research Institute, Plano TX, United States;
[22] Medstar Health, Washington D.C., United States;
[23] Allegheny-Singer Research Institute, Pittsburgh PA, United States;
[24] The Ohio State University, Columbus OH, United States;
[25] VU University Medical Center, Amsterdam, The Netherlands;
[26] Mayo Clinic Florida, Jacksonville FL, United States;
[27] Washington University, St. Louis MO, United States;
[28] Golden Jubilee National Hospital, Glasgow, United Kingdom;
[29] Sheffield Pulmonary Vascular Disease Unit, Royal Hallamshire Hospital, Sheffield, United Kingdom;
[30] University of Newcastle, Newcastle, United Kingdom;
[31] Department of Molecular Medicine, University of Pavia, Pavia, Italy;
[32] Fondazione IRCCS Policlinico San Matteo, Pavia, Italy;
[33] INSERM UMR_S 1219, Bordeaux Population Health Research Center, University of Bordeaux, France; Department of Neurology, Bordeaux University Hospital, Bordeaux, France;
[34] Department of Medicine at Intermountain Medical Center and the University of Utah, Murray UT, United States;
[35] University of Cincinnati, Cincinnati OH, United States;
[36] Duke University Medical Center, Durham NC, United States;
[37] Institute of Clinical Molecular Biology, University of Kiel, Kiel, Germany;
[38] Mayo Clinic, Rochester MN, United States;
[39] Weill Cornell Medical College and The Houston Methodist Hospital, Houston TX, United States;
[40] Department of Medicine and Arizona Health Sciences Center, University of Arizona, Tucson, AZ, United States;
[41] CAGE, Cincinnati Children's Hospital Medical Center, Department of Pediatrics, University of Cincinnati College of Medicine, Cincinnati OH, United States;
[42] Tufts Medical Center, Boston MA, United States;
[43] Cincinnati Children's Hospital Medical Center, Cincinnati OH, United States;
[44] Children's Hospital Colorado, University of Colorado Denver, Aurora CO, United States;
[45] Rhode Island Hospital, Providence RI, United States;
[46] Ludwig Boltzmann Institute for Lung Vascular Research, Graz, Austria;
[47] Medical University of Graz, Graz, Austria;
[48] Indiana University, Indianapolis IN, United States;

[49] Department of Internal Medicine 1, University of Kiel, Kiel, Germany;
[50] Institute of Medical and Biomedical Education, St George's University of London, London, United Kingdom;
[51] Royal United Hospitals Bath NHS Foundation Trust, Bath, United Kingdom;
[52] Division of Biomedical Informatics, Cincinnati Children’s Hospital Medical Center, Department of Pediatrics, University of Cincinnati College of Medicine, Cincinnati OH, United States;
[53] Great Ormond Street Hospital, London, United Kingdom;
[54] Inova Heart and Vascular Institute, Falls Church VA, United States;
[55] LA Biomedical Research Institute at Harbor-UCLA, Torrance CA, United States;
[56] East Carolina University, Greenville NC, United States;
[57] Vanderbilt University Medical Center, Nashville TN, United States;
[58] Department of Medicine, Vanderbilt University School of Medicine, Nashville, TN, United States;
[59] Departments of Biomedical Informatics and Pharmacology, Vanderbilt University School of Medicine, Nashville, TN, United States;
[60] Columbia University, New York NY, United States;
[61] Wayne State University, Detroit MI, United States;
[62] University Hospital of Cleveland, Cleveland OH, United States;
[63] Boston University School of Medicine, Boston MA, United States;
[64] University of Pittsburgh, Pittsburgh PA, United States;
[65] Papworth Hospital, Papworth, United Kingdom;
[66] Department of Clinical Genomics, Ambry Genetics, Aliso Viejo, CA, United States;
[67] University of Minnesota, Minneapolis MN, United States;
[68] UT Southwestern, Dallas TX, United States;
[69] LSU Health, Shreveport LA, United States;
[70] University of Rochester Medical Center, Rochester NY, United States;
[71] Spectrum Health Hospitals, Grand Rapids MI, United States;
[72] Seattle Children's Hospital, Seattle WA, United States;
[73] Department of Infection, Immunity & Cardiovascular Disease, University of Sheffield, Sheffield, United Kingdom;
[74] US Department of Veterans Affairs Medical Center, Cincinnati, Ohio, USA;
[75] State Key Laboratory of Respiratory Diseases, Guangzhou Institute of Respiratory Health, The First Affiliated Hospital of Guangzhou Medical University, Guangzhou, Guangdong, China. 51018
