# Supplementary material for: Mendelian randomisation and experimental medicine approaches to interleukin-6 as a drug target in pulmonary arterial hypertension
Source: Eur Respir J. 2022 Mar 10;59(3):2002463. doi: 10.1183/13993003.02463-2020 (PMC8907935; doi:10.1183/13993003.02463-2020)
Supplement: Supplementary file 2 [file ERJ-02463-2020.Shareable.pdf]

EUROPEAN RESPIRATORY JOURNAL
ORIGINAL RESEARCH ARTICLE
M. TOSHNER ET AL.

SHAREABLE PDF

Mendelian randomisation and experimental medicine approaches to interleukin-6 as a drug target in pulmonary arterial hypertension

Mark Toshner [1,2,20], Colin Church[3,20], Lars Harbaum[4], Christopher Rhodes[4], Sofia S. Villar Moreschi[5], James Liley[1,5], Rowena Jones[1], Amit Arora[6], Ken Batai[7], Ankit A. Desai[8], John G. Coghlan[9], J. Simon R. Gibbs[4], Dee Gor[10], Stefan Gräf [1], Louise Harlow[2], Jules Hernandez-Sanchez[10], Luke S. Howard [4], Marc Humbert [11], Jason Karnes[6], David G. Kiely [12], Rick Kittles[6], Emily Knightbridge[1], Brian Lam[13], Katie A. Lutz[14], William C. Nichols[14], Michael W. Pauciulo[14], Joanna Pepke-Zaba[2], Jay Suntharalingam[15], Florent Soubrier [16], Richard C. Trembath[17], Tae-Hwi L. Schwantes-An[8], S. John Wort[4], Martin R. Wilkins [4], Sean Gaine[18], Nicholas W. Morrell[1,21] and Paul A. Corris[19,21], the Uniphy Clinical Trials Network

[1]Dept of Medicine, University of Cambridge, Cambridge, UK. [2]Royal Papworth Hospital, Cambridge, UK. [3]Golden Jubilee Hospital, Glasgow, UK. [4]Heart Lung Research Institute, Imperial College, London, UK. [5]MRC Biostatistical Unit, University of Cambridge, Cambridge, UK. [6]Dept of Epidemiology and Biostatistics, University of Arizona, Tucson, AZ, USA. [7]Dept of Urology, University of Arizona, Tucson, AZ, USA. [8]Dept of Medicine, Indiana University, Indianapolis, IN, USA. [9]Royal Free Hospital, London, UK. [10]Roche Products Limited, Welwyn Garden City, UK. [11]Université Paris-Sud, Le Kremlin-Bicêtre, Paris, France. [12]Royal Hallamshire Hospital, Sheffield, UK. [13]Institute of Metabolic Sciences, University of Cambridge, Cambridge, UK. [14]Division of Human Genetics, Cincinnati Children's Hospital Medical Center, Cincinnati, OH, USA. [15]Royal United Hospital, Bath, UK. [16]Sorbonne Universités, INSERM, Paris, France. [17]Genetics and Molecular Medicine, King's College, London, UK. [18]Mater Misericordiae University Hospital, Dublin, Ireland. [19]Dept of Medicine, Newcastle University, Newcastle, UK. [20]Authors contributed equally to this work. [21]Authors contributed equally to this work.

Corresponding author: Mark Toshner (mrt34@medschl.cam.ac.uk)

Check for updates

Shareable abstract (@ERSpublications)
Tocilizumab did not block IL-6 signalling in pulmonary arterial hypertension. Multicentre mendelian randomisation studies additionally did not demonstrate evidence for IL-6R in pulmonary arterial hypertension. https://bit.ly/3xkDxS5

Cite this article as: Toshner M, Church C, Harbaum L, *et al*. Mendelian randomisation and experimental medicine approaches to interleukin-6 as a drug target in pulmonary arterial hypertension. *Eur Respir J* 2022; 59: 2002463 [DOI: 10.1183/13993003.02463-2020].

This single-page version can be shared freely online.

Copyright ©The authors 2022.

This version is distributed under the terms of the Creative Commons Attribution Licence 4.0.

Received: 23 June 2020
Accepted: 15 July 2021

Abstract

Background Inflammation and dysregulated immunity are important in the development of pulmonary arterial hypertension (PAH). Compelling preclinical data supports the therapeutic blockade of interleukin-6 (IL-6) signalling.

Methods We conducted a phase 2 open-label study of intravenous tocilizumab ($8\ mg \cdot kg^{-1}$) over 6 months in patients with group 1 PAH. Co-primary end-points were safety, defined by incidence and severity of adverse events, and change in pulmonary vascular resistance. Separately, a mendelian randomisation study was undertaken on 11 744 individuals with European ancestry including 2085 patients with idiopathic/heritable disease for the IL-6 receptor (*IL6R*) variant (rs7529229), known to associate with circulating IL-6R levels.

Results We recruited 29 patients (male/female 10/19; mean±SD age 54.9±11.4 years). Of these, 19 had heritable/idiopathic PAH and 10 had connective tissue disease-associated PAH. Six were withdrawn prior to drug administration; 23 patients received at least one dose of tocilizumab. Tocilizumab was discontinued in four patients owing to serious adverse events. There were no deaths. Despite evidence of target engagement in plasma IL-6 and C-reactive protein levels, both intention-to-treat and modified intention-to-treat analyses demonstrated no change in pulmonary vascular resistance. Inflammatory markers did not

predict treatment response. Mendelian randomisation did not support an effect of the lead *IL6R* variant on risk of PAH (OR 0.99, p=0.88).

Conclusion Adverse events were consistent with the known safety profile of tocilizumab. Tocilizumab did not show any consistent treatment effect.
